# *Origanum vulgare* ssp. *vulgare*: Chemical Composition and Biological Studies

**DOI:** 10.3390/molecules23082077

**Published:** 2018-08-19

**Authors:** Ilioara Oniga, Cristina Pușcaș, Radu Silaghi-Dumitrescu, Neli-Kinga Olah, Bogdan Sevastre, Raluca Marica, Ioan Marcus, Alexandra Cristina Sevastre-Berghian, Daniela Benedec, Carmen Elena Pop, Daniela Hanganu

**Affiliations:** 1 Department of Pharmacognosy, Iuliu Hatieganu University of Medicine and Pharmacy, 12 I. Creanga Street, Cluj-Napoca 400010, Romania; ioniga@umfcluj.ro (I.O.); dhanganu@umfcluj.ro (D.H.); 2 Department of Chemistry and Chemical Engineering, Babes-Bolyai University, 11 A. Janos Street, Cluj-Napoca 400028, Romania; cbischin@chem.ubbcluj.ro (C.P.); rsilaghi@chem.ubbcluj.ro (R.S.-D.); 3 Faculty of Medicine, Pharmacy and Dental Medicine, Vasile Goldiş Western University of Arad, 86 L. Rebreanu Street, Arad 310414, Romania; neliolah@yahoo.com; 4 Department of Paraclinics, University of Agricultural Sciences and Veterinary Medicine, 3-5 Mănăştur Street, Cluj-Napoca 400372, Romania; bogdan.sevastre@usamvcluj.ro (B.S.); raluca.vidrighinescu@gmail.com (R.M.); ioan.marcus@usamvcluj.ro (I.M.); 5 Department of Physiology, Iuliu Hatieganu University of Medicine and Pharmacy, 6 L. Pasteur Street, Cluj-Napoca, 400349, Romania; alexandra_berghian@yahoo.com; 6 Department of Drug Industry and Biotechnology, Iuliu Hatieganu University of Medicine and Pharmacy, 12 I. Creanga Street, Cluj-Napoca 400010, Romania

**Keywords:** *Origanum vulgare*, polyphenols, cythocrome c, antioxidant, antimicrobial, hepatoprotective

## Abstract

The biological properties and main phenolic compounds of the *O. vulgare* L. ssp. *vulgare* extract are described in the present paper. The polyphenolic compounds were analyzed by chromatographic and spectrophotometric techniques. The antioxidant potential was evaluated using several methods: CUPRAC (cupric ion reducing antioxidant capacity), FRAP (ferric reducing ability of plasma), inhibition of lipid peroxidation catalyzed by cytochrome *c,* and superoxide (SO) scavenging assays. The antimicrobial activity of the oregano extract was evaluated by means of agar-well diffusion assay. The hepatoprotective effect of the *O. vulgare* extract on CCl_4_-induced hepatotoxicity was evaluated in rats. Liver injury was estimated by determination of alanine aminotransaminase (ALT), aspartate aminotransaminase (AST), gamma-glutamyl transferase GGT, total protein and albumin concentrations, glutathione peroxidase (GPx), catalase (CAT), superoxide dismutase (SOD) and malondialdehyde (MDA). These values were improved by the administration of oregano extract. A specific phenolic profile was evidenced by these data, with large amounts of rosmarinic and chlorogenic acids. The oregano extract showed very strong antioxidant activity in good agreement with the phenolic content. Antimicrobial activity was good, especially against *Salmonella enteritidis* and *Aspergillus niger* strains. The high hepatoprotective, antioxidant and antimicrobial activity, along with polyphenol-rich content, can support the use of *O. vulgare* in therapy. We also expect our results to open new research directions for designing important new drug products, using indigenous plant material.

## 1. Introduction

*Origanum vulgare* L. (oregano, wild marjoram), an herbaceous Mediterranean species of the *Lamiaceae* family, comprises several subspecies such as *hirtum* (Link) Ietsw., *vulgare* L., *viridulum* (Martrin-Donos) Nyman, *glandulosum* (Desfontaines) Ietswaart, *gracile* (Koch) Ietsw., *virens* (Hoffmanns. & Link) Ietsw., and *viride* L. [1,2]. In Romania, the most widespread *O. vulgare* species is *O. vulgare* ssp. *vulgare* [1,3]. In terms of chemical composition, research on *O. vulgare* species from various countries have previously been reported: Italy [4], Greek [5,6], Serbia [7], Egypt [8], Pakistan [9,10], China [10], India [11], Iraq [12], Macedonia [13]. Representatives of the following important classes of active principles were revealed: essential oil (with carvacrol and/or thymol, linalool, and *p*-cymene), polyphenols (flavonoids and phenolic acids), triperpenoids, and sterols [8,14,15]. European oregano has been traditionally used since ancient times for its carminative, stomachic, emmenagogue, and expectorant effects to treat cramps, flatulence, coughs, or menstrual problems [2,15]. *Origanum vulgare*’s longstanding use in traditional medicine attracts even greater interest in designing new pharmaceutical formulations in various areas [2]. New investigations have shown many therapeutic properties: antimicrobial [12], antiviral [10], antioxidant [10], anti-inflammatory [15], antispasmodic [2], antiurolithic [9], antiproliferative [4,15], neuroprotective [16], etc. Because of its high antioxidant activity, *O. vulgare* is an important natural source for the preservation of different food or cosmetics [2,15,17]. At present, interest in this plant is increasing not only in other countries, but also in Romania, especially as an important therapeutic alternative. Previously research on Romanian *O. vulgare* used plant material from different sources: Iasi county [18], culture from Arges county [16], or commercial samples [19]. The *O. vulgare* subspecies was not notified. Therefore, by focusing on *Origanum vulgare* ssp. *vulgare*, from Cluj county spontaneous flora, this study was conducted to determine the main polyphenols and to assess the biological potential as antioxidant, antimicrobial and hepatoprotective. Knowledge about native oregano is important to obtain good quality natural products, rich in active principles, with a well determined composition, which could be recommended in different diseases, in efficient doses.

## 2. Results and Discussion

### 2.1. Chromatographic Analysis of Phenolic Compounds

HPLC analysis of the *O. vulgare* ssp. *vulgare* extract revealed the presence of several phenolic acids as well as flavonoids. Identification of the compounds was based on their retention times, and UV and MS spectra as compared to standards. Quantification was performed using an external standard method, with 21 standard phenolic compounds: 10 phenolic acids and 11 flavonoids. After analysis, 10 of them were confirmed and determined in this work: four phenolic acids (gentisic, chlorogenic, *p*-coumaric and rosmarinic acids) and six flavonoids (hyperoside, isoquercitrin, rutin, quercitrin, quercetin and luteolin) (Table 1). The concentrations of the identified polyphenolic compounds (mg polyphenol/g dry plant material) in the analyzed sample are presented in Table 1. The most abundant phenolic compound was rosmarinic acid (12.83 mg/g), followed by chlorogenic acid (2.10 mg/g), while the other two phenolic acids (gentisic and *p*-coumaric acids) were found in trace amounts. Other authors reported similar or higher amounts of rosmarinic acid [19,20,21,22], while, for chlorogenic acid, the results were inferior to those obtained by us [19,20]. Regarding flavonoids, four glycosides (hyperoside, isoquercitrin, rutin and quercitrin) and two aglycones of flavonoids (quercetin and luteolin) were found. Hyperoside was found in the largest amount (1.05 mg/g), followed by isoquercitrin (0.71 mg/g), rutin (0.64 mg/g) and quercitrin (0.50 mg/g). Among flavonoid aglycons, luteolin was quantified (0.10 mg/g), and quercetin was only identified. Rutin and quercetin were also found in *O. vulgare* from Greece [6]. Other authors also reported several other flavonoids in *O. vulgare* extracts (apigenin, naringenin, cirsimartin, diosmetin, etc.) and phenolic acids (syringic, protocatechuic, homovanillic, hydroxybenzoic, caffeic acids, etc.) [16,18]. A more detailed comparison with previous reports was very difficult to achieve because the authors used different extraction methods, different experimental protocols, and plant materials from different geographical areas. Nevertheless, this research brings novelty and originality as regards the phenolic composition of the Romanian species *O. vulgare* ssp. *vulgare*. We identified gentisic, chlorogenic, *p*-coumaric and rosmarinic acids, hyperoside, isoquercitrin, quercitrin, quercetin and luteolin. *O. vulgare* can be considered an important source of polyphenolic compounds (in particular, rosmarinic acid). Our results can be useful to obtain extracts with a good chemical characterization, important for further experiments or clinical trials, to extend the therapeutic potential of this species.

### 2.2. Polyphenolic Content and Antioxidant Activity of *O. vulgare* ssp. *vulgare* Extract 

Total polyphenol content (TPC) was determined spectrophotometrically using the Folin–Ciocalteu assay, and was expressed as mg gallic acid equivalent (GAE)/g of dry plant material. Total flavonoid content was determined by the aluminum chloride colorimetric method, and the result was expressed as mg rutin equivalent (RE)/g of dry plant material. The content of total caffeic acid derivatives in the ethanolic extract was analyzed spectrophotometrically, and the results were expressed as mg of caffeic acid equivalents (CAE)/g of dry plant material. All experiments were performed in triplicate (Table 2). 

Our results (Table 2) showed that *O. vulgare* ssp. *vulgare* contains many phenolic compounds: total polyphenols (94.69 mg/g) and caffeic acid derivatives (29.92 mg/g). These results are similar to those reported for plants harvested in Macedonia [13], Germany [14,17], as well as higher than some other authors’ determinations [16,18]. Regarding the content of flavonoids, we obtained lower values (38.46 mg/g) than those obtained for Macedonian species [13], but close to other results for Romanian species [16].

In addition, the extract of *O. vulgare* was tested for its antioxidant activity using several in vitro models: ferric and cupric reducing powers, inhibition of lipid peroxidation catalyzed by cytochrome *c*, and antioxidant capacity against superoxide radicals. The results obtained are presented in Table 2. The oregano extract demonstrated a strong cupric and ferric ion reducing antioxidant capacity (1284 µM TE/g, and 794.40 µM TE/g, respectively), in good agreement with the polyphenolic content. Our findings appear to be well supported by several previous studies [13,15,16]. Thus, aerial parts collected from the Romanian *O. vulgare* can be a rich source of phenolic compounds with a strong antioxidant activity that qualifies them as a potential functional food with beneficial effects for maintaining and promoting health, as well as for reducing the risk of some pathological processes. The antioxidant reactivity of the *O. vulgare* extract against superoxide was significant.

**Figure 1:**
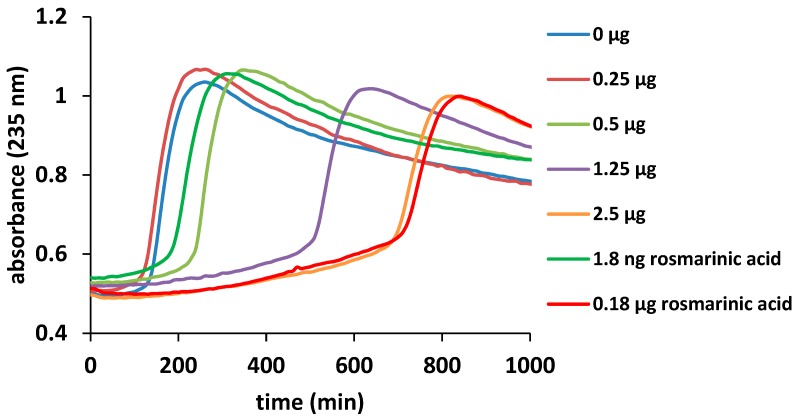
Liposome oxidation by cytochrome *c*, in the presence of *O. vulgare* ssp. *vulgare* extract and rosmarinic acid in the concentration shown in the figure legend.

A very good antioxidant capacity was also obtained with the inhibition of liposome oxidation by cytochrome *c* (Figure 1). This method, described in detail in [23] and proposed as a more relevant physiological antioxidant method for testing the antioxidant capacity of the natural extracts, is based on the capacity of cytochrome *c* or other heme proteins to induce the oxidation of lipids, in this case, arranged in liposomes. The sigmoidal curves shown in Figure 1 monitor the absorbance at 235 nm (wavelength specific for lipids oxidation) over time, and the inflection points are taken as a measure of the length of the lag phase. The antioxidant capacity of the extract is directly correlated with the increases in the values of the inflection points. Thus, for the control samples, the value is 159 ±12 min, very close to the value obtained for 0.25 µg/mL extract (146 ± 13 min). With the increases in the concentration of *O. vulgare* extract, clear increases of the lag phase are observed. Thus, for 0.5, 1.25 and 2.5 µg extract, the values for the inflection points are 265 ± 1.00, 593 ± 89.00 and 812 ± 152.00, respectively. These results are comparable with those observed for the capacity of rosmarinic acid to inhibit lipid oxidation at the concentration shown in the Figure 1; particularly, 2.5 μg of extract are seen to have the same effect as 0.18 μg rosmarinic acid. This is in good agreement with the fact that HPLC measurements revealed 12 mg/g rosmarinic acid in the extracts, while TPC measurements revealed a total of 95 mg/g phenolics, hence suggesting that rosmarinic acid is the most important antioxidant compound, in accordance with its highest concentration between the polyphenols. 

### 2.3. Determination of Antimicrobial Activity

The evaluation of antimicrobial activity of the *O. vulgare* extract is recorded in Table 3 and Table 4. The extract was effective in suppressing microbial growth and it showed different diameters of inhibition zones that were compared to those of the standards: gentamicin for antibacterial activity and amphotericin B for antifungal activity (Table 3). The extract was an inhibitor against all tested bacteria, with a good antimicrobial activity (inhibition diameter between 16 and 19 mm). Our findings are comparable to results obtained by other authors who revealed a moderate to good antibacterial activity of oregano extract [21,24,25,26]. Regarding the activity on *A. niger*, our sample exhibited a good antifungal effect (inhibition diameter of 19 mm). Few tests were carried out on *Aspergillus* fungi, particularly *A. niger*, and the *O. vulgare* extract notably inhibited their growth [26]. In terms of MIC, the same bacterial and fungal strains were used. The results in Table 4 show a variation of MIC values from 19.53 to 156.25 mg/mL (Table 4); the highest activity was found against *A. niger* fungus (19.53 μg/mL). These results are consistent with those obtained by the diffusion method.

### 2.4. Hepatoprotective Activity of *O. vulgare* ssp. *vulgare* Extract

**Figure 2:**
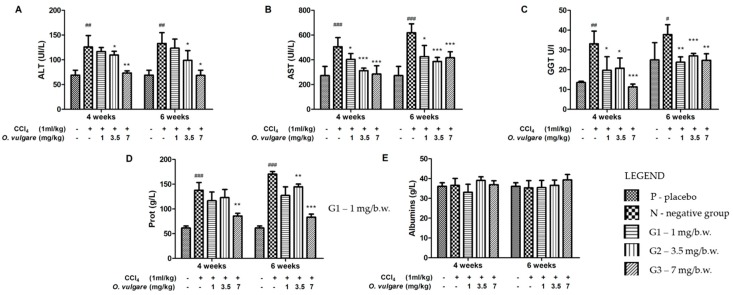
Effects of *O. vulgare* ssp. *vulgare* extract on: ALT (**A**); AST (**B**); GGT (**C**) activity and plasma total protein (**D**); and albumin (**E**) concentration (mean ± SD, 5 animals/group). “+” is the group which received CCl_4_ and “-” is the group which didn’t received CCl_4_. ^#^ *p* < 0.05, ^##^ *p* < 0.01, and ^###^ *p* < 0.001 compared to the reference group; * *p* < 0.05, ** *p* < 0.01, and *** *p* < 0.001 compared to the CCl_4_ alone treated group.

The effect of the *O. vulgare* extract on CCl_4_-induced hepatotoxicity was investigated in normal and hepatotoxic rats. Alanine aminotransaminase (ALT), aspartate aminotransaminase (AST), Gamma-glutamyl transferase (GGT), and total protein and albumin concentrations in plasma; and glutathione peroxidase (GPx), catalase (CAT), superoxide dismutase (SOD) and malondialdehyde (MDA) in liver tissue were estimated to assess the extent of liver injury. All animals survived up to the end of the protocol without clinical signs of disease. The body weight gain and food consumption remained close to normal in all experimental groups. Mice receiving CCl_4_ alone exhibited elevated activity of plasma transaminases, ALT, AST, snf GGT, as compared to the reference group (Figure 2), suggesting hepatocellular injury. Albumin levels remained similar to those of the reference group, implying that liver destruction was not extensive enough to induce liver insufficiency. However, total proteins were elevated, based on the globulin fraction, most likely due to inflammatory liver injury. *O. vulgare* therapy alleviates liver destruction, manifesting by a reduction of plasma transaminase activity and a reduction of globulin levels (Figure 2). The highest dose seems to provide the best protection, but no clear dose-related effect could be found.

**Figure 3:**
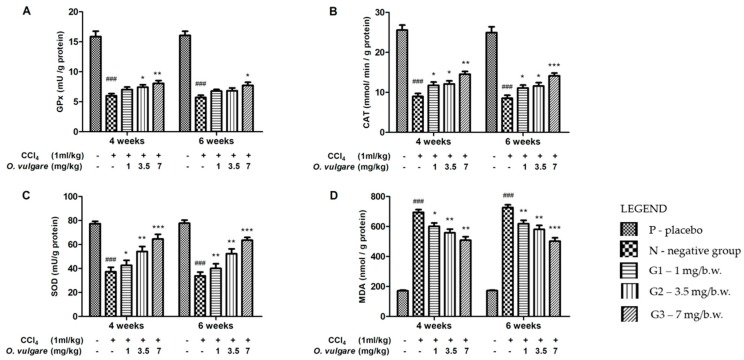
Effects of *O. vulgare* ssp. *vulgare* extract on: glutathione peroxidase (GPx) (**A**); catalase (CAT) (**B**); superoxide dismutase (SOD) (**C**); and malondialdehyde (MDA) (**D**) (mean ± SD, five animals/group). “+” is the group which received CCl_4_ and “-” is the group which didn’t received CCl_4_. ^###^ *p* < 0.001 compared to the reference group; * *p* < 0.05, ** *p* < 0.01, and *** *p* < 0.001 compared to the CCl_4_ alone treated group.

The influence of *O. vulgare* extract therapy on liver oxidative stress markers is displayed in Figure 3. Expectedly, administration of CCl_4_ induced severe oxidative stress expressed by the inhibitory activity of antioxidant enzymes such as glutathione peroxidase (GPx), catalase (CAT), superoxide dismutase (SOD) and by a threefold increase of lipid peroxidation. The intensity of oxidative stress was almost the same at both four and six weeks. The protective effect of *O. vulgare* extract therapy was visible at the lowest dose, but the dose of 7 mg/b.w. (body weight) proved to be the most effective. The protection was clearly visible at both four and six weeks. *O. vulgare* extract therapy could partially restore the activity of antioxidant enzymes and to alleviate lipid peroxidation. 

**Figure 4:**
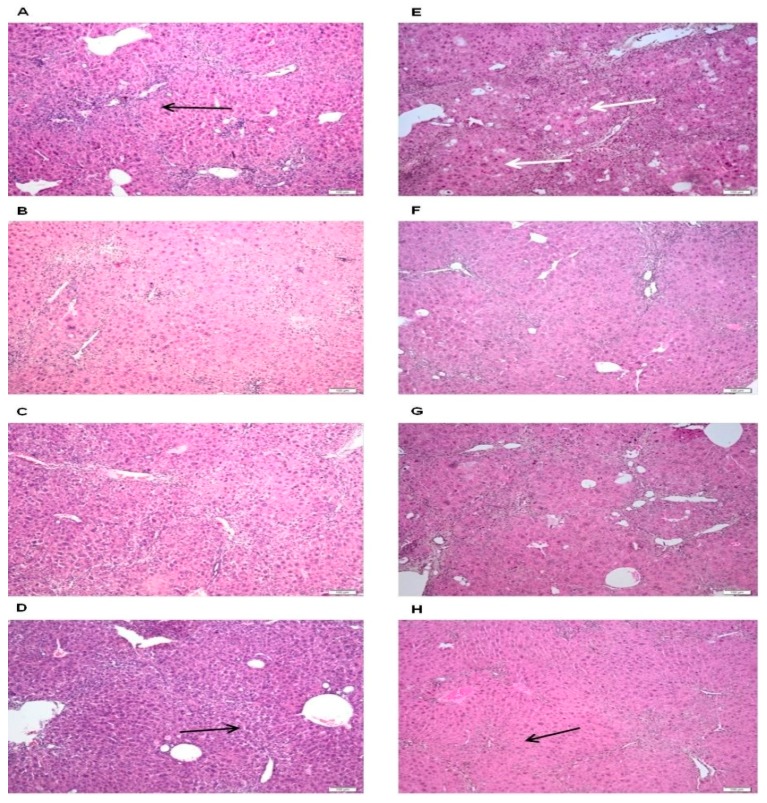
Effects of *O. vulgare* ssp. *vulgare* extract on the histologic aspect of the liver: (**A**,**E**) the negative control groups receiving only carbon tetrachloride (1 mL/b.w.) showed infiltrate around the portal spaces (black arrow), necrotic foci (white arrows); and the groups receiving therapy with the extract in a dose of: 1 mg/b.w., (**B**,**F**); 3.5 mg/b.w.; (**C**,**G**); and 7 mg/b.w. (**D**,**H**). The animals treated with the highest dose showed decreased fibrosis (black arrows). Duration: (**A**–**D**) four weeks; (**E**–**H**) six weeks. Hematoxylin and eosin stain; Bar, 100 μm.

In both negative control groups (those that received only CCl_4_), we observed a considerable variety of inflammatory, degenerative and necrotic lesions, confirming the previous findings in plasma biochemistry and liver oxidative stress markers. The inflammatory infiltrate was predominantly present in the periportal spaces and mainly consisted of mononuclear cells (Figure 4A,D). Hepatocellular necrosis was also identified primarily in the perilobular region. Among the tested groups, there were differences regarding the intensity of the lesions. Thus, in the animals receiving the extract at dose of 1 mg/b.w., the extent of parenchymal injuries was similar to that observed in the control group. The loss of architecture, hepatocyte necrosis and the inflammatory infiltrate were accompanied by the presence of clear vacuoles in the cytoplasm (hepatic degeneration). This aspect was more visible after eight weeks (Figure 4B,F). In the groups that received the average dose of *O. vulgare* extract (3.5 mg/b.w.), the examination revealed fibrous tissue proliferation, at both six and eight weeks. Portal fibrosis is an atypical reparatory mechanism following a considerable loss of hepatocytes and inflammatory response (Figure 4C,G). The protective effect of the plant extract was best observed at the highest dose: inflammation in the portal region, as well as fibroplasia and hepatic necrosis decreased. After eight weeks of administration, hepatocyte regeneration was supported by an increased number of mitotic cells, while cellular and nuclear polymorphism was weak to moderate (Figure 4D,H). Our findings are in line with those of previous studies investigating the hepatoprotective effect of *O. vulgare* extract. The efficiency of an *O. vulgare* aqueous extract was already tested using the CCl_4_ toxicity rat model [27] and similar to our findings, the *O. vulgare* therapy effectively improved oxidative stress parameters, preventing the extent of liver injury. However, the protective effect of the aqueous extract was visible at 50 mg/b.w., a significantly higher dose than the doses used by us. The alcohol extraction method, used to prepare our extract, is likely to better extract and preserve the polyphenols (flavonoids and caffeic acid derivatives) which are responsible for the antioxidant effect. In the present study, we obtained comprehensive results demonstrating that the *O. vulgare* ssp. *vulgare* alcoholic extract can alleviate the extent of liver damage in mice models of CCl_4_ toxic hepatitis. *O. vulgare* therapy reduces the plasma transaminase activity and the severity of microscopic liver lesions. Presumably, hepatoprotection is mainly provided by its antioxidant properties, as mice receiving therapy also showed lower oxidative damage levels and improved antioxidant enzyme activities.

## 3. Materials and Methods 

### 3.1. Plant Material 

The aerial parts of *O. vulgare* ssp. *vulgare* (Voucher No. 96) were harvested from Beliș (46°41′4″ N 23°1′45″ E), Cluj county, Romania, during the flowering period. The plant material was identified by Dr. Ilioara Oniga (“Iuliu Haţieganu” University of Medicine and Pharmacy Cluj-Napoca).

### 3.2. Chemicals 

Ferulic, sinapic, gentisic, gallic, rosmarinic acids, patuletin, luteolin from Roth (Karlsruhe, Germany), cichoric, and caftaric acids were purchased from Dalton (Toronto, ON, Canada). Chlorogenic acid, caffeic acid, *p*-coumaric acid, rutin, isoquercitrin, quercitrin, hyperoside, myricetol, fisetin, quercetin, apigenin, and kaempferol were acquired from Sigma (St. Louis, MO, USA). Sodium nitrite, sodium molybdate, ammonium acetate, sodium hydroxide, sodium carbonate, carbon tetrachloride, neocuproine (2,9-dimethyl-1,10-phenanthroline), 2,4,6-tris(2-pyridyl)-s-triazine (TPTZ), Tris-HCl buffer, copper chloride, ferric chloride, Trolox (6-hydroxy-2,5,7,8-tetramethylchroman-2-carboxylic acid), isoflurane, buffered formalin solution paraffin wax, and hematoxylin–eosin stain were purchased from Sigma-Aldrich (Steinheim, Germany). HPLC grade methanol, acetic acid, analytical grade orthophosphoric acid, hydrochloric acid, aluminum chloride, sodium acetate, ethanol, nitroblue tetrazolium chloride (NBT) and Folin–Ciocalteu reagent were purchased from Merck (Darmstadt, Germany). DPPH (2,2-diphenyl-1-picrylhydrazyl), β-nicotinamide adenine dinucleotide reduced disodium salt, and phenazine methosulfate were obtained from Alfa-Aesar (Karlsruhe, Germany). CAT, XOD and GPx assay kits were purchased from BioVision, USA. Liposomes were obtained by suspending 5 mg/mL soybean lecithin (Alfa Aesar, Karlsruhe, Germany) in phosphate buffer followed by sonication and horse heart purified cytochrome *c* from Sigma-Aldrich (Steinheim, Germany). Plasma biochemistry was performed on blood drawn on clot activator vacutainers; the blood was centrifuged and serum samples were stored at −20 °C until used. Serum chemistry was measured using a screen point semi-automatic analyzer (STAT-FAX 1904 Plus Global Medical Instrumentation Inc. 6511 Bunker Lake Blvd. Ramsey Minnesota, 55303), using special kits and following the producer specifications (Diachem, Hungary). All microorganism products were distributed by MicroBioLogics^®^: *Staphylococcus aureus* ATCC 6538P (Gram-positive bacteria), *Listeria monocytogenes* ATCC 13932 (Gram-positive bacteria), *Escherichia coli* ATCC 25922 (Gram-negative bacteria), *Salmonella enteritidis* ATCC 13076 (Gram-negative bacteria) and one fungal strain, *Aspergillus niger* ATCC 16888. The spectrophotometric data were acquired using a Jasco V-530 UV-vis spectrophotometer (Jasco International Co., Ltd., Tokyo, Japan).

### 3.3. Preparation of *O. vulgare* ssp. *vulgare* Extract

Ten grams of powdered vegetal product were extracted with 20 mL of 70% ethanol in an ultrasonic bath (Polsonic Palczynski Sp. J., Warsaw, Poland, Sonic 3), at 60 °C and sonicated for 30 min. The extract was filtered through paper filters in 20 mL flasks and then centrifuged (2422 g) for 20 min, and the supernatants were recovered [28]. 

### 3.4. HPLC Chromatographic Conditions and Instrumentation

Analysis was performed on an Agilent 1100 HPLC Series system (Agilent, Santa Clara, CA, USA) equipped with G1322A degasser, G13311A binary gradient pump, column thermostat, G1313A autosampler, and G1316A UV detector that was coupled to an Agilent 1100 mass spectrometer. HPLC-MS analysis of the studied extracts was performed according to a previously validated and described method [28].

Separation of the compounds was carried out on a reverse-phase analytical column (Zorbax SB-C18 100 × 3.0 mm i.d., 3.5 μm particle). Detection was performed using UV and MS with an electrospray ion source in negative mode. The chromatographic data were processed using the ChemStation and DataAnalysis software from Agilent. The mobile phase, with methanol and acetic acid 0.1% (*v*/*v*), was used in a binary gradient. For 35 min, the elution was performed with a linear gradient, starting at 5% methanol and finishing at 42% methanol. The flow rate of the mobile phase was 1 mL/min. Five microliters were used for injection. For qualitative analysis only, the MS signal was used. The standard MS spectra were integrated in a mass spectra library. Under these chromatographic conditions the couples—caftaric with gentisic acids and caffeic with chlorogenic acids—could not be quantitatively determined due to overlapping. Hence, these four carboxylic acids were determined only based on MS spectra, whereas for the rest of the compounds, the linearity of the calibration curves was very good (R^2^ > 0.998), with detection limits in the range of 18–92 ng/mL. The detection limits were calculated as the minimal concentration yielding a reproducible peak with a signal-to-noise ratio greater than three. The analyses were performed using an external standard method; retention times were determined with a standard deviation ranging from 0.04 min to 0.19 min. Accuracy was between 94.13% and 105.3% for all substances. The compounds in the *O. vulgare* extract were identified by comparison of their retention times and recorded electrospray mass spectra with those of the standards under the same conditions.

### 3.5. Determination of Polyphenols Content

Total polyphenolic content was determined according to the European Pharmacopoeia, using the Folin–Ciocalteu method, with a calibration curve of gallic acid (R^2^ = 0.999), and the results were expressed as mg of gallic acid equivalent (GAE)/g dry plant material [29]. Two milliliters of *O. vulgare* extract were diluted 25 times, then mixed with 1.0 mL of Folin–Ciocalteu reagent, 10.0 mL of distilled water and diluted to 25.0 mL with a sodium carbonate solution (290 g/L). Absorbance was measured at 760 nm, after 30 min.

A spectrophotometric method, based on flavonoid-aluminum chloride (AlCl_3_) complexation, was employed to determine total flavonoid content. In brief, 5.0 mL extract were mixed with 5.0 mL of sodium acetate (100 g/L), 3.0 mL of aluminum chloride 25 g/L, and filled up to 25 mL by methanol in a calibrated flask. The mixture was allowed to stand for 15 min and absorbance was measured at 430 nm. Using a calibration curve (R^2^ = 0.999), the result was expressed as mg rutin equivalent (RE) per g dry plant material [30]. 

The caffeic acid derivatives content was determined according to the Romanian Pharmacopoeia (10th Edition, *Cynarae folium* monograph), using Arnows’ reagent (10.0 g sodium nitrite and 10.0 g sodium molybdate made up to 100 mL with distilled water). The percentage of phenolic acids, expressed as caffeic acid equivalent on dry plant material (mg CAE/g), was determined using an equation that was obtained from a calibration curve based on caffeic acid (R^2^ = 0.994) [30]. 

### 3.6. Determination of Antioxidant Properties of *O. vulgare* Extract

To investigate the in vitro antioxidant properties of the oregano extract, several methods were used: FRAP (the ferric reducing ability of plasma), CUPRAC (modified cupric reducing antioxidant capacity), inhibition of lipid peroxidation catalyzed by cytochrome *c*, and superoxide scavenging reactivity.

#### 3.6.1. CUPRAC (Cupric Reducing Antioxidant Capacity) Assay

This method assesses the color of a copper complex with the neocuproine (2, 9-dimethyl-1, 10-phenanthroline). The reduction of the copper ion (II) to the copper ion (I) determines a color change from light green to reddish-orange. The antioxidant capacity by the CUPRAC method was determined at 450 nm [31]. To 1.0 mL of 7.5 mM neocuproine solution, 1.0 mL 10 mM copper chloride solution and 1.0 mL ammonium acetate buffer (pH = 6.8) were added. This mixture is CUPRAC reagent. To 4.0 mL from extract, up to 1.1 mL and 3.0 mL CUPRAC reagent were added. The mixture was incubated at room temperature and absorbance was measured at 450 nm after 30 min. A blank solution was prepared in the same manner using water instead of sample. The calibration curve was plotted using concentrations in the range of 11.4–45.6 g/L Trolox standard (y = 0.0148x + 0.0112; R^2^ = 0.9733) [32].

#### 3.6.2. FRAP (Ferric Reducing Antioxidant Power) Assay

The FRAP method relies on the change in the color of a complex with Fe^+3^ ion of the 2,4,6-tri(2-pyridyl)-1,3,5-triazine (TPTZ) radical by the reduction of the ferric ion to the ferrous ion (Fe^+2^) in this complex [33]. To 2.5 mL of 10 mM TPTZ solution in 40 mM HCl, 2.5 mL 20 mM ferric chloride solution and 25 mL acetate buffer (pH = 3.6) were added. This mixture is the FRAP reagent. To 4.0 mL extract up to 0.8 mL water and 6.0 mL FRAP reagent were added. until a blank solution was prepared in the same manner using water instead of sample. Trolox was used as a reference. The color change was correlated with the antioxidant capacity by measuring absorbance at 450 nm. Using a calibration curve (R^2^ = 0.992), the result was converted to µM Trolox equivalents/100 mL extract.

#### 3.6.3. Inhibition of Lipid Peroxidation Catalyzed by Cytochrome *c*

The liposomes were obtained from 5.0 mg lecithin dissolved in 10 mM phosphate buffer, pH 7 and sonicated for 20 min in an ultrasonic bath. Thirty microliters of liposomes were mixed with 2.0 µM cytochrome *c* and 5.0 µL of different extract concentration (50, 100, 250, 400 and 500 µg/mL). The experiment was monitored at 235 nm, a wavelength specific for lipid oxidation [23]. The experimental data were fitted with a Boltzmann curve for the calculation of the inflection point.

#### 3.6.4. Superoxide Radical (SO) Scavenging Activity Assay

Superoxide radical is a major biological source of reactive oxygen species. Even if the superoxide anion is a weak oxidant, it can generate strong and dangerous hydroxyl radicals as well as singlet oxygen, and significantly contributes to oxidative stress. The superoxide anion radicals were generated in 2.0 mL of Tris-HCl buffer (16 mM, pH 8.0), with 2.0 mL of nitroblue tetrazolium (NBT, 0.3 mM) and 2.0 mL nicotinamide adenine dinucleotide solution (NADH, 0.936 mM). To the mixture was added 0.4 mL extract diluted to 4.0 mL with water. The reaction was initiated by adding 2.0 mL phenazine methosulfate solution (PMS, 0.12 mM) and the mixture was incubated at 250 °C for 5 min. Absorbance was measured at 560 nm against a blank sample prepared from 2.0 mL Tris-HCl buffer, with 2.0 mL NBT and 2.0 mL NADH solution, 4.0 mL water and 2.0 mL PMS solution. As standard, 4.0 mL of 1.152 mg/mL Trolox solution was used. The standard solution was added to 2.0 mL Tris-HCl buffer, with 2.0 mL NBT solution and 2.0 mL NADH solution, and finally, 2 mL PMS solution were added (0.12 mM) [34].

### 3.7. Determination of Antimicrobial Activity

The *O. vulgare* ethanolic extract was investigated for antimicrobial activity using an agar well diffusion method. Agar plates were inoculated with a standardized inoculum of the test microorganisms: *S. enteritidis*, *E. coli*, *L. monocytogenes*, *S. aureus*, and *A. niger*. Each microorganism was suspended in Mueller–Hinton (MH) broth and diluted to approximately 10E6 colony forming unit (CFU)/mL. They were “flood-inoculated” onto the surface of MH agar and MH Dextrose Agar (MDA) and then dried. Six-millimeter diameter wells were cut from the agar using a sterile cork-borer, and 60 μL of each extract were delivered into the wells. The plates were incubated at 37 °C and the diameters of the growth inhibition zones were measured after 24 h. All tests were performed in triplicate, and clear halos greater than 10 mm were considered as positive results. Gentamicin and amphotericin B were used as standard drugs. The negative control was 70% ethanol (diameter = 6 mm) [35]. 

The minimum inhibitory concentrations (MICs) of the extract were determined by an agar dilution method including the same strains of microorganisms as used in the agar disk diffusion method [33]. For this experiment, 100 µL nutrient broths were placed in a 96-well plate and 100 µL of plant extract were added in the first ten lines. Then, 100 µL were aspirated from every well and placed in the second well line of the plate. This technique was used to obtain the desired dilutions up to line 10; from the last well, 100 µL mixes were discharged as follows: 50.0 µL, 25.0, 12.5, 6.25, 3.12, 1.56, 0.78, 0.39, 0.19, and 0.09 µL of plant extracts in 100 µL medium. Each well was seeded with 5.0 µL of a 24 h bacterial culture suspension, adjusted to be similar to 0.5 McFarland scale 10^8^ CFU/mL), and incubated for 16–24 h (48 h for fungi) at 35 ± 2 °C. MIC was detected by the lowest concentration of the analyzed product in which the development of the bacterium strain was inhibited (the medium remained clear). The negative control (70% ethanol) concentration for the determination of MIC using the serial dilution method was similar to the concentration used in the 1st well of the plate [36].

### 3.8. Hepatoprotective Activity Method

Throughout the entire project, the mice were housed in standard polypropylene cages, at optimum density and in standard laboratory conditions (temperature 25 ± 1 °C, relative humidity 55 ± 5%, and 12 h light/dark cycle). They were allowed free access to standard granular diet and water. Housing conditions and all the procedures performed on laboratory animals complied with the European Directive 22.09.2010/63/EU on the protection of animals used for scientific purposes, as well as with national legislation. According to the national law 43/2014 on the protection of animals used for scientific purposes, the project was approved by the Commission for Bioethics and Research Ethics of UASVM (accord No. 68/30.05.2017), and the Veterinary Sanitary and Food Safety Authority (project authorization No. 73/14.06.2017). Prior to the assessment of the hepatoprotective effect, the *O. vulgare* extract was subjected to toxicity testing following the OECD Guidelines for the Testing of Chemicals, Test No. 425: Acute Oral Toxicity: Up-and-Down Procedure. Five female Swiss mice were included, and all of them survived, showing no signs of toxicity at a dose of 2000 mg/b.w. administered orally. Additionally, fourteen days later, at the end of the toxicity test, gross necropsy showed no alteration in the internal organs—a finding further supported by liver and kidney histological examination [37]. 

The main study was conducted on fifty female Swiss mice, aged six months, with a mean body weight of 32 ± 2.63 g. First, they were divided into five equal groups as follows. Mice receiving only placebo treatment were the reference group. The second group (negative control) received carbon tetrachloride (CCl_4_) in a dose of 1.0 mL/kg diluted in vegetable oil. CCl_4_ was administrated orally three times a week, on Days 1, 3 and 5, up to the end of the protocol, using a lubricated catheter; furthermore, these animals received only placebo therapy instead of extract. The other three groups received the same dose of CCl_4_, on the same days, but they benefited from the *O. vulgare* extract therapy in dose of 1, 3.5, and 7 mg/b.w., respectively. The animals received the extract three times a week, on the same days, but four hours later, also up to the end of the study. Each of the five groups of ten mice was further divided into two equal subgroups; one was euthanized four weeks after the beginning of the study, while the remaining animals were allowed to live for another two weeks. Before use, the alcoholic extract was maintained into a rotary evaporator until the alcohol was removed, and then it was given immediately to the animals.

The body weight was measured initially, then weekly, until the end of the study. Food and water intake in all groups of mice were also monitored daily. Finally, all animals were subjected to deep narcosis using isoflurane, and blood samples were collected from the orbital sinus. Later, the animals were euthanized by prolonged narcosis; they were considered dead when no heart and respiratory activity was recorded. The irreversibility of the phenomenon was assured by cervical dislocation. 

Fresh K EDTA samples were used for hematology; while for plasma biochemistry the blood was drawn in clot activator vacutainers. The blood was allowed to clot, then centrifuged (1465 g) for 15 min. The serum was stored at −20 °C and thawed just before it was used for biochemical analysis. 

#### Oxidative Stress Markers

Extraction of total protein from liver tissue was done by homogenization with a phosphate buffer solution (at 10 mM, pH 7.4). The obtained protein extracts were analyzed for total protein content, catalase (CAT), superoxide dismutase (SOD) activity, glutathione peroxidase (GPx) activity, and lipid peroxidation. The activity of CAT, XOD and GPx was determined using the corresponding assay kits (BioVision, USA), according to the manufacturer’s specifications. The concentration of malonyl dialdehyde (MDA) was determined using reaction with thiobarbituric acid (TBA). The results were measured by a METERTECH Spectrophotometer SP-830 Plus.

For histopathological examination, the tissue fragments were first placed in 10% formalin solution for 24 hours, processed for paraffin embedding and cut to a thickness of 5–6 μm. The slides were than stained with the standard histological hematoxylin-eosin stain. The microscopic image was obtained using the Olympus BX 41 microscope. Image acquisition was performed using an Olympus UC30 digital camera and OLYMPUS Stream Basic software. Histopathological examination aimed to identify the main degenerative, necrotic and inflammatory processes present in the liver, at different stages of the pathological process and at different extract doses.

### 3.9. Statistical Analysis 

The samples were analyzed in triplicate or more; the average and the relative standard deviations were calculated using the Excel software package. The Gaussian distribution was checked by the Shapiro–Wilk normality test. Statistical analyses were performed by two-way ANOVA followed by Bonferroni post-test. Statistical significance was at *p* < 0.05 (95% confidence interval). Statistical values and figures were obtained using GraphPad Prism version 5.0 for Windows, GraphPad Software, San Diego, CA, USA.

## 4. Conclusions

This study provides data on the chemical and biological characterization of the spontaneous Romanian *Origanum vulgare* ssp. *vulgare*. In the ethanolic extract of oregano, four phenolic acids (gentisic, chlorogenic, *p*-coumaric and rosmarinic acids) and six flavonoids (hyperoside, isoquercitrin, rutin, quercitrin, quercetin and luteolin) were identified by HPLC-MS. The biological effects, particularly the antioxidant, antimicrobial and hepatoprotective, were significant. The antioxidant activity, evaluated using CUPRAC, FRAP, inhibition of lipid peroxidation catalyzed by cytochrome *c*, and SO scavenging assays, indicated that *O. vulgare* extract had a high antioxidant potential, in line with the total polyphenolic content. This antioxidant property could partially explain the assessed hepatoprotective activity through a reduction of plasma transaminase levels and of the severity of microscopic liver lesions in the animals. The oregano extract revealed a good antimicrobial activity against the tested bacterial and fungal strains. Taken together, these results reinforce the importance of this millennial species and open new perspectives for the research and design of new herbal medicines, obtained from indigenous vegetal material.

## Figures and Tables

**Table 1:** Phenolic compounds determined by the HPLC method in *O. vulgare* ssp. *vulgare* extract.

Compounds	[M − H]^−^, *m*/*z*	Retention Time (t_R_), min	UV Detection	MS Detection	Concentration (mg/g)
Gentisic acid	153	3.69 ± 0.04	NO	YES	<0.02
Chlorogenic acid	353	6.43 ± 0.05	YES	YES	2.10 ± 0.14
*p*-Coumaric acid	163	9.48 ± 0.08	NO	YES	<0.02
Hyperoside	463	18.60 ± 0.12	YES	YES	1.05 ± 0.03
Isoquercitrin	463	20.29 ± 0.10	YES	YES	0.71 ± 0.19
Rutin	609	20.76 ± 0.15	YES	YES	0.64 ± 0.15
Rosmarinic acid	360	21.80 ± 0.10	YES	YES	12.83 ± 2.19
Quercitrin	447	23.64 ± 0.13	YES	YES	0.50 ± 0.08
Quercetin	301	27.55 ± 0.15	NO	YES	<0.02
Luteolin	285	29.64 ± 0.19	YES	YES	0.10 ± 0.04

Values are the mean ± SD (n = 3).

**Table 2:** Total polyphenol content and antioxidant activity of *O. vulgare* ssp. *vulgare* extract.

Sample	TPC (mg GAE/g)	Flavonoid (mg RE/g)	Caffeic Acids (mg CAE/g)	CUPRAC (µM TE/g)	FRAP (µM TE/g)	SO Scavenging (µM TE/g)
*O. vulgare*	94.69 ± 4.03	38.46 ± 3.54	29.92 ± 1.08	1284 ± 66	794.40 ± 25.80	44.00 ± 0.56

Each value is the mean ± SD of three independent measurements. TPC, total polyphenols content; SO, superoxide; GAE, gallic acid equivalents; RE, rutin equivalents; CAE, caffeic acid equivalents; TE, Trolox equivalents.

**Table 3:** Antimicrobial activity of *O. vulgare* ssp. *vulgare* extract.

Sample	Diameter of Inhibition Zone (mm)				
	*S. enteritidis*	*E. coli*	*L. monocytogenes*	*S. aureus*	*A. niger*
*O. vulgare* ssp. *vulgare*	18.0 ± 0.00	16.0 ± 0.00	17.0 ± 1.00	16.0 ± 1.00	19.0 ± 0.00
Gentamicin	19.0 ± 1.00	18.0 ± 1.06	22.0 ± 0.50	18.0 ± 0.00	-
Amphotericin B	**-**	-	-	-	21.0 ± 0.00

The values represent the average of three determinations ± SD.

**Table 4:** Minimum inhibitory concentration (MIC) values of *O. vulgare* ssp. *vulgare* extract.

Samples	MICs (µg/mL)				
	*S.* *enteritidis*	*E. coli*	*L.* *monocytogenes*	*S. aureus*	*A. niger*
*O. vulgare* ssp. *vulgare*	78.13	156.25	156.25	78.13	19.53
70% Ethanol	625	2500	1250	625	2500
